# Impaired autophagy: a link between neurodegenerative and neuropsychiatric diseases

**DOI:** 10.1111/jcmm.12349

**Published:** 2014-08-19

**Authors:** Mira Polajnar, Eva Žerovnik

**Affiliations:** Department of Biochemistry and Molecular and Structural Biology, Jožef Stefan InstituteLjubljana, Slovenia

**Keywords:** autophagy, protein aggregation, neurodegenerative diseases, progressive myoclonus epilepsies, psychiatric diseases

## Abstract

Protein misfolding, and subsequent aggregation have been proven as the leading cause of most known dementias. Many of these, in addition to neurodegeneration, show profound changes in behaviour and thinking, thus, psychiatric symptoms. On the basis of the observation that progressive myoclonic epilepsies and neurodegenerative diseases share some common features of neurodegeneration, we proposed autophagy as a possible common impairment in these diseases. Here, we argue along similar lines for some neuropsychiatric conditions, among them depression and schizophrenia. We propose that existing and new therapies for these seemingly different diseases could be augmented with drugs used for neurodegenerative or neuropsychiatric diseases, respectively, among them some which modulate or augment autophagy.

## Protein aggregation is present in neurodegenerative and neuropsychiatric disorders

Protein folding in cells is normally maintained by the action of chaperones. When a mutation or cellular stress occurs [*e.g*. acidification, increased temperature, increased reactive oxygen species (ROS) generation, hypoxia], proteins can misfold and aggregate.

Protein aggregation and amyloid fibril formation are at the core of many neurodegenerative diseases (NDs) [1]. The original amyloid cascade hypothesis [2] stated that the amyloid plaques formed by amyloid beta (Aβ) peptide deposit in brain cause a cascade of events leading to the observed pathology of Alzheimer's disease (AD). However, later work showed that intracellular soluble oligomers and prefibrillar aggregates may be the actual neurotoxic agents. The severity of the cognitive decline in AD correlated with levels of oligomers in the brain, rather than the total Aβ plaque burden [3].

Protein aggregates share a common mechanism of damage to neurons and glia. This includes impairment of mitochondria [4], slowing of axonal transport [5] and oxidative stress [6]. Amyloid toxicity is suggested to derive from the interaction of oligomers with membranes and formation of channels (pores) [7].

Variations in Disrupted in Schizophrenia 1 (DISC1) gene is implicated in major neuropsychiatric disorders (NPs), such as schizophrenia, bipolar disorder, depression and autism. DISC1 is expressed in neuronal dendritic spines and controls spine and synapse development [8]. It has a dual role in both progenitor proliferation and post-mitotic neuronal migration that are regulated through (de)phosphorylation [9]. DISC1 forms dimers, octamers, higher oligomers and insoluble aggregates in some chronic psychiatric diseases [10,11]. It was recently published that the formation of DISC1 aggregates [12] led to a decrease of available soluble protein and a gain in toxic function by affecting axonal mitochondrial transport [13]. Furthermore, a S704 mutant associated with major depression and schizophrenia has a higher tendency to form aggregates [14] and another rare sequence variant, R37W, was recently shown to directly impair anterograde mitochondrial transport [15]. Furthermore, oxidative stress was proposed to contribute to various cognitive impairments in a transgenic DISC1 mouse model [16].

One of many toxic molecular mechanisms of protein aggregation is sequestration of chaperones. Chaperones normally act as an effective first line of defence against misfolded proteins. Unfolded or misfolded proteins thus should not present a problem for healthy neurons or other cells. Namely, regulatory function of molecular chaperones is tightly connected with other pathways of protein degradation if this protective mechanism fails. Misfolded proteins and aggregates are cleared from the cell by either ubiquitin–proteasome system (UPS) or autophagy.

## Role of the UPS and autophagy in NDs

Sequestration and degradation of soluble oligomers and aggregates of misfolded proteins by the UPS and autophagy are a well-regulated pro-survival response. Whereas autophagy is mainly responsible for the degradation of long-lived proteins and is largely non-specific, the UPS is used for rapid and specific degradation of proteins, mediated by polyubiquitination [17].

In autophagy, cytoplasmic components, whole organelles, viruses and proteins are delivered to the lysosomal compartment for degradation. Autophagy has been mainly described as non-selective degradative pathway induced by starvation; however, autophagy is needed more than just for nutrient management [18]. One of the main functions of autophagy is to enforce intracellular quality control by selective disposal of protein aggregates and damaged organelles (for review see [19]). In fact, ablation of two genes essential for autophagy, *atg5* and *atg7*, leads to accumulation of ubiquitin-positive aggregates and progressive loss of neurons in mice [20,21].

Macroautophagy (hereafter referred to as autophagy) starts with engulfing a portion of the cytoplasm surrounded by an isolation membrane into a cup-shaped phagophore, eventually forming a new vacuole known as an autophagosome. Next step is fusion of the autophagosome with lysosome. This step can be inhibited by dysfunction in the lysosomal pathway and/or the susceptibility of the lysosome to oxidative stress. When cells are overwhelmed by ROS accumulation, the resulting oxidative stress can additionally destroy mitochondrial integrity and lead to apoptosis.

The UPS and autophagy are complementary in their mode of action [22]. When an increased accumulation of misfolded proteins overwhelms the UPS, ubiquitinated, misfolded proteins are transported to the nucleus where they form aggresomes, which are predominantly cleared by autophagy [23,24]. Many NDs are characterized by an accumulation of ubiquitinated misfolded protein deposits, pointing to a failure of UPS to clear them. At this stage, misfolded proteins accumulated in aggresomes should be further degraded by autophagy that is less specific than the UPS. On the other hand, in several NDs, autophagic vacuoles (autophagosomes or autophagolysosomes) accumulate, suggesting lysosomal dysfunction. One of the dynein motor proteins was shown to be essential for autophagy and protein clearance [25]. Direct impairment of autophagy was recently observed in Parkinsons's disease (PD) and amyotrophic lateral sclerosis (ALS) upon inhibition or knockdown of dyneins respectively [26,27]. Similarly, it was recently suggested that the impairment of the microtubule transport in an Alzheimer's disease (AD) *Drosophil*a model is because of defective phosphorylation of the tau protein [28]. On the other hand, in Huntington's disease (HD), it seems that the physiological rate of autophagic turnover remains intact; however, the cells are unable to recognize the cytosolic deposits [29].

We propose that cellular stress in the form of ROS or general metabolic imbalance/acidification could cause proteins to misfold and aggregate. This would in turn diminish or overwhelm degradation and chaperone machineries. Because of autophagy and mitophagy impairment, cells would be unable to clear aggregates and damaged organelles. Additional mitochondrial dysfunction, excitotoxicity and pore formation lead to increased intracellular Ca^2+^ levels, which is a characteristic feature of both necrosis and apoptosis.

## Possible entry points for targeting autophagy

Autophagy is regulated by intracellular and extracellular signals mediated by at least two complexes: (1) Atg1/unc-51-like kinase (ULK) complex that acts downstream of the mammalian target of rapamycin (mTOR) complex 1 (mTORC1) and (2) Beclin 1/class III phosphatidylinositol 3-kinase (PI3K) complex. mTORC1 (a polyprotein complex that contains mTOR Ser/Thr kinase) regulates cell growth, transcription, translation and autophagy [30], is inhibited by starvation, *i.e*. amino acids, growth factors, the cellular AMP:ATP ratio and by calcium signalling.

Because the signalling pathways of autophagy are very complex and still not fully understood, there are many potential entry points where the pathways could be modulated. Rapamycin, one of the best known inducers of autophagy through mTOR inhibition, has shown promise in a fruit fly model of HD [31] and other proteinopathies like PD [32] and in AD [33] and prion [34,35] mouse models (for review see [36]). Furthermore, caloric restriction and resveratrol both promote Sirtuin1 (SIRT1)-dependent autophagy, which was shown to be neuroprotective in models of AD and ALS [37]. Resveratrol was additionally shown to reduce oxidative stress and neuronal cell death [38] and protect from Aβ neurotoxicity in a rat model [39]. Interestingly, acetylase inhibitor spermidine stimulated autophagy independent of SIRT1. In spite of the difference in the primary targets of resveratrol and spermidine, both agents activate convergent pathways and elicit similar changes in the (de)acetylation pattern of proteins [40]. Spermidine was found to reduce neuron loss in ALS mouse model [41] and improve memory in a HD rodent model [42]. Trehalose is another autophagy-inducing component acting independently of the mTOR pathway. It has shown promise in models of HD [43], ALS [44], tauopathies [45,46] and prion disease [47]. It was recently reported that astemizole, a drug already approved for human use, inhibited pathological prion protein (PrPSc) replication and induced autophagy through an unknown mechanism [48].

On the other hand, it was reported that a mouse ALS model presented exacerbated apoptosis of neuronal cells and disease progression after treatment with rapamycin [35]. Specific pathogenesis of the disease and the fact that rapamycin is primarily used as an immunosuppressant may underlie the devastating effects on neurons as other previously mentioned autophagy inducers improved cell survival in ALS models. This also points out the importance of finding the right target of the complex autophagic machinery.

One should also be aware of counterproductive effects of autophagy boosting in specific neurodegenerative cases. This especially holds true in cases when an important component of the autophagic process is missing downstream of the activation complexes. In this case, boosting autophagic activation may lead to an increased accumulation of non-degradable autophagosomes and even contribute to the aetiology of the disease.

## Autophagy impairment is shared between NDs, PMEs and neuropsychiatric diseases

Neurodegenerative diseases, whose frequency increases with ageing, place a considerable burden on Western societies. Whereas NDs such as AD and PD are in most cases sporadic and are associated with multiple gene abnormalities, progressive myoclonus epilepsies (PMEs) [49] are monogenic, familial syndromes. PMEs, among them Unverricht–Lundborg disease (EPM1) and Lafora disease (EPM2), are a group of different genetic generalized epilepsies with myoclonic and tonic–clonic seizures, dementia and progressive neurodegeneration of grey matter [50].

On the basis of the observation that PMEs and NDs share common features of neurodegeneration, we previously proposed autophagy as such a possible common impairment [51]. Here, we argue along similar lines for some NPs, among them depression and schizophrenia.

Overlapping disease signs between NDs and NPs can be observed in both human patients and animal models. Several neuropsychiatric comorbidities like depression and apathy have been observed in patients with NDs [51]. Abnormalities in social behaviour have been observed also in mouse models of PD [52], AD [53] and HD [54]. Diseases like schizophrenia and bipolar disorder are considered more neurodevelopmental and the neurodegenerative hypothesis is still controversial [55,56], although schizophrenic mouse models are known to exhibit thinning of the cerebral cortex [57].

It was recently reported that there is a reduction in Beclin 1, one of the key proteins in autophagy, mRNA levels in the hippocampus of schizophrenia patients [58]. Additionally, a novel neurological and psychiatric disorder was recently described and termed Beta-propeller Protein Associated Neurodegeneration (BPAN). Patients are described with intellectual disability, depression disorders, epileptic seizures and parkinsonism. The underlying cause of BPAN is a mutation in the WD repeat containing protein 45 (*WDR45*) gene, coding for WIPI4 protein [59]. WIPI4 belongs to the WD-repeat protein Interacting with Phosphoinosides (WIPI) family that is important for autophagosomal membrane formation.

An antidepressant activity of rapamycin was reported in an animal model, although the authors proposed other intracellular interactions besides mTOR inhibition contributed to the effect [60]. Similarly, antidepressant-like effects were observed in mice with manic-like behaviours after treatment with trehalose [61].

Lithium, a classic mood stabilizer, was proposed for treatment of HD [62], ALS [63], and was shown to reduce PrPSc through the induction of autophagy [64]. Together with two other mood-stabilizing and anticonvulsant drugs, valproic acid (VPA) and carbamazepam (CBZ), lithium was suggested to indirectly induce autophagy through inhibiting inositol monophosphatase (IMPA1) and other enzymes in the phosphatidylinositol pathway [65,66]. It was shown that lithium and valproate have a synergistic neuroprotective effect in an ALS mouse model [67] and the effect of lithium was matched with increased expression of phosphoinositide phosphatase PTEN, a positive regulator of autophagy [68]. Unfortunately, several follow-up studies on mice and patients found no significant effect of lithium on the ALS disease pathology (reviewed in [69]). On the other hand, other studies show effects of lithium and CBZ in mouse models of tauopathy [70] and AD [71].

## A link between Wnt and mTOR signalling and link to neurodegenerative and neuropsychiatric diseases

**Fig. 1:**
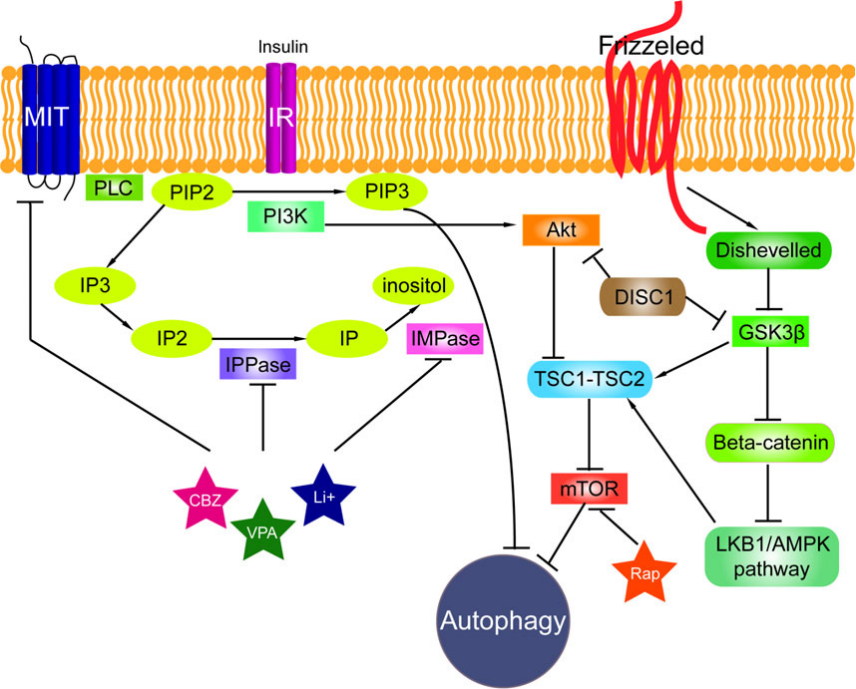
The signalling pathways connected to autophagy. Phosphatidylinositol signalling pathway is regulated by Class I phosphoinositide 3-kinases (PI3Ks), which are activated by kinase receptors like insulin receptors (IR) and responsible for the production of phosphatidylinositol (3,4,5)-triphosphate (PIP3) from phosphatidylinositol 4,5-bisphosphate (PIP2). Phospholipase C (PLC) cleaves the PIP2 into diacyl glycerol and inositol 1,4,5-trisphosphate (IP3). Inositol polyphosphate 1-phosphatase (IPPase) catalyses inositol bisphosphate (IP2) to inositol monophosphate (IP), which is further dephosphorylated by inositol monophosphatase (IMPase) to inositol. High-affinity inositol transport is additionally catalysed by the active myo-inositol/H+ transporter (MIT). MIT, IPPase and IMPase are all inhibited by carbamazepine (CBZ), valproic acid (VPA) and lithium (Li+). PI3Ks have been linked to an extraordinarily diverse group of cellular functions through regulation of the Akt/TSC1-TSC2/mTOR pathway. Disrupted in Schizophrenia 1 (DISC1) in its wild-type form also negatively regulates both GSK3β and Akt (also known as Protein Kinase B, PKB). Tuberous sclerosis protein 1 and 2 (TSC1/2) form a complex that like rapamycin (Rap) inhibits mammalian target of rapamycin kinase (mTOR). Wnt signalling activation is mediated through binding of a Wnt-protein ligand to a Frizzled family receptor, which passes the biological signal to the protein Dishevelled (Dsh). Dsh negatively regulates glycogen synthase kinase-3 beta (GSK3β), which alternatively inhibits β-catenin, one of the central proteins of the Wnt signalling pathway. β-catenin, however, negatively regulates LKB1/AMPK pathway (liver kinase B1/5' adenosine monophosphate-activated protein kinase) that indirectly regulates autophagy.

Wnt and mTOR signalling pathways are tightly linked to autophagy, which is their common downstream event (Fig. 1).

Wnt signalling is involved in the development of the brain and spinal cord and in the extension of numerous subpopulations of sensory and motor neurons. However, some central nervous system (CNS)-related diseases in adulthood have also been associated with components of the Wnt signalling pathway, highlighting the fundamental role of this pathway in the proper functioning of the mature CNS. Recently, it was shown that both autophagy and proteasome-mediated degradation negatively regulate Wnt signalling by promoting degradation of Dishevelled (Dsh), a centre mediator in Wnt signalling pathway [72]. Dsh inhibits glycogen synthase kinase 3 beta (GSK3β), which, when active, inhibits β-catenin – another important mediator of the Wnt pathway. β-catenin was shown to be negatively regulated by FIP200, a known positive regulator of autophagy, through ubiquitination [73]. β-catenin is also a negative regulator of the LKB1/AMPK pathway that, when activated, suppresses mTOR signalling [74]. Furthermore, Wnt also activates mTOR signalling through inhibition of GSK3β. When GSK3β is active, it phosphorylates tuberous sclerosis protein 2 (TSC2) and suppresses mTOR [75].

DISC1 was shown to determine the proliferation and fate of neural progenitors by stabilizing β-catenin through a direct interaction with GSK3β and regulation of β-catenin activity [76]. On the other hand, DISC1 suppression in neurons led to overactivation of Akt signalling. This effect could be prevented by mTOR inhibition, a downstream target of Akt [77]. Of note, DISC1 loss of function as a result of oligomerization and aggregation is implied in schizophrenia and some other neuropsychiatric diseases.

Although activation of Wnt signalling has been proven as beneficial in cell and animal models [78–80], conflicting results are being reported. For example, different modes of cytotoxicity have been suggested for Aβ. Firstly, an antagonistic effect of Aβ on the canonical Wnt/β-catenin signalling was reported [81] and just recently, other authors reported a positively regulated non-canonical Wnt/PCP (planar cell polarity) signalling in a Aβ-based mouse model [82].

Nevertheless, data from other ND models suggest that there is, in fact, an increase in activation of the canonical Wnt pathway. Evidence for a direct interaction between the PD-associated protein parkin (ubiquitin E3 ligase) and β-catenin has been published; increased levels of total and active (dephosphorylated) β-catenin were found in mice lacking parkin. This increase in Wnt/β-catenin signalling resulted in an increase in dopaminergic (DA) neuron proliferation and death, suggesting that a decrease in the degradation of β-catenin may lead to loss of DA neurons as they try to re-enter the cell cycle [83].

Mutations in malin, an E3 ubiquitin ligase, are associated with the appearance of the severest type of PME, Lafora disease. It was recently shown that a decrease in malin levels significantly increased the levels of Dsh and up-regulated Wnt signalling. Overexpression of malin enhanced the degradation of Dsh through K48- and K63-linked ubiquitination that are linked to both proteasome and autophagy degradation [84].

Mutations in Prickle-1 are associated with a form of progressive myoclonic epilepsy, similar to EPM1. Prickle is part of the non-canonical Wnt/PCP pathway and was shown to interact with RE1-silencing transcription factor, an essential regulator of neuronal genes. Depleting Prickle-1 gene in the zebra fish embryo altered the convergent extension movements essential for gastrulation and disrupted normal calcium signalling [85]. Furthermore, Prickle-1 was capable of lowering Dsh *in vitro* and negatively regulated Wnt/β-catenin pathway by promoting Dsh degradation through ubiquitination in liver cancer (Prickle-1 was also found to be underexpressed in human hepatocellular carcinoma) [86].

## Concluding remarks

1. We propose that an impaired autophagy could be involved in neuropsychiatric diseases similarly to NDs, such as AD, PD, ALS and prion diseases. A vicious circle may perpetuate: autophagy is compromised, protein aggregates accumulate and eventually overload cellular degradation and transport systems. These impairments could also explain an increase in oxidative stress.
2. It was shown for major mental disorders involving DISC1 that they have increased protein aggregation and oxidative stress was shown.
3. In accordance, augmenting autophagy could be beneficial for several proteinopathies and neuropsychiatric conditions. Indirect evidence suggests that in addition to lithium, anticonvulsant drugs and mood stabilizers such as CBZ and VPA both positively influence mood and stimulate autophagy. In the light of recent findings that DISC1, protein implicated in major NPs, forms aggregates these drugs could also be used to augment cognition in schizophrenia and depression.

## References

[1] Irvine GB, El-Agnaf OM, Shankar GM (2008). Protein aggregation in the brain: the molecular basis for Alzheimer's and Parkinson's diseases. Mol Med.

[2] Hardy JA, Higgins GA (1992). Alzheimer's disease: the amyloid cascade hypothesis. Science.

[3] Lue LF, Kuo YM, Roher AE (1999). Soluble amyloid beta peptide concentration as a predictor of synaptic change in Alzheimer's disease. Am J Pathol.

[4] Rhein V, Song X, Wiesner A (2009). Amyloid-beta and tau synergistically impair the oxidative phosphorylation system in triple transgenic Alzheimer's disease mice. Proc Natl Acad Sci USA.

[5] Stokin GB, Lillo C, Falzone TL (2005). Axonopathy and transport deficits early in the pathogenesis of Alzheimer's disease. Science.

[6] Butterfield DA, Bush AI (2004). Alzheimer's amyloid beta-peptide (1-42): involvement of methionine residue 35 in the oxidative stress and neurotoxicity properties of this peptide. Neurobiol Aging.

[7] Lashuel HA, Hartley D, Petre BM (2002). Neurodegenerative disease: amyloid pores from pathogenic mutations. Nature.

[8] Duan X, Chang JH, Ge S (2007). Disrupted-In-Schizophrenia 1 regulates integration of newly generated neurons in the adult brain. Cell.

[9] Ishizuka K, Kamiya A, Oh EC (2011). DISC1-dependent switch from progenitor proliferation to migration in the developing cortex. Nature.

[10] Leliveld SR, Bader V, Hendriks P (2008). Insolubility of disrupted-in-schizophrenia 1 disrupts oligomer-dependent interactions with nuclear distribution element 1 and is associated with sporadic mental disease. J Neurosci.

[11] Leliveld SR, Hendriks P, Michel M (2009). Oligomer assembly of the C-terminal DISC1 domain (640-854) is controlled by self-association motifs and disease-associated polymorphism S704C. Biochemistry.

[12] Atkin T, Kittler J (2012). DISC1 and the aggresome: a disruption to cellular function?. Autophagy.

[13] Atkin TA, Brandon NJ, Kittler JT (2012). Disrupted in Schizophrenia 1 forms pathological aggresomes that disrupt its function in intracellular transport. Hum Mol Genet.

[14] Korth C (2012). Aggregated proteins in schizophrenia and other chronic mental diseases: DISC1opathies. Prion.

[15] Ogawa F, Malavasi EL, Crummie DK (2014). DISC1 complexes with TRAK1 and Miro1 to modulate anterograde axonal mitochondrial trafficking. Hum Mol Genet.

[16] Johnson AW, Jaaro-Peled H, Shahani N (2013). Cognitive and motivational deficits together with prefrontal oxidative stress in a mouse model for neuropsychiatric illness. Proc Natl Acad Sci USA.

[17] McCray BA, Taylor JP (2008). The role of autophagy in age-related neurodegeneration. Neurosignals.

[18] Mizushima N, Yamamoto A, Matsui M (2004). *In vivo* analysis of autophagy in response to nutrient starvation using transgenic mice expressing a fluorescent autophagosome marker. Mol Biol Cell.

[19] Mizushima N, Levine B, Cuervo AM (2008). Autophagy fights disease through cellular self-digestion. Nature.

[20] Hara T, Nakamura K, Matsui M (2006). Suppression of basal autophagy in neural cells causes neurodegenerative disease in mice. Nature.

[21] Komatsu M, Waguri S, Chiba T (2006). Loss of autophagy in the central nervous system causes neurodegeneration in mice. Nature.

[22] Nedelsky NB, Todd PK, Taylor JP (2008). Autophagy and the ubiquitin-proteasome system: collaborators in neuroprotection. Biochim Biophys Acta.

[23] Corboy MJ, Thomas PJ, Wigley WC (2005). Aggresome formation. Methods Mol Biol.

[24] Kopito RR (2000). Aggresomes, inclusion bodies and protein aggregation. Trends Cell Biol.

[25] Batlevi Y, Martin DN, Pandey UB (2010). Dynein light chain 1 is required for autophagy, protein clearance, and cell death in Drosophila. Proc Natl Acad Sci USA.

[26] Cai ZL, Shi JJ, Yang YP (2009). MPP+ impairs autophagic clearance of alpha-synuclein by impairing the activity of dynein. NeuroReport.

[27] Ikenaka K, Kawai K, Katsuno M (2013). dnc-1/dynactin 1 knockdown disrupts transport of autophagosomes and induces motor neuron degeneration. PLoS ONE.

[28] Ambegaokar SS, Jackson GR (2012). The downward spiral of tau and autolysosomes: a new hypothesis in neurodegeneration. Autophagy.

[29] Martinez-Vicente M, Talloczy Z, Wong E (2010). Cargo recognition failure is responsible for inefficient autophagy in Huntington's disease. Nat Neurosci.

[30] Schmelzle T, Hall MN (2000). TOR, a central controller of cell growth. Cell.

[31] Ravikumar B, Vacher C, Berger Z (2004). Inhibition of mTOR induces autophagy and reduces toxicity of polyglutamine expansions in fly and mouse models of Huntington disease. Nat Genet.

[32] Xiong N, Jia M, Chen C (2011). Potential autophagy enhancers attenuate rotenone-induced toxicity in SH-SY5Y. Neuroscience.

[33] Majumder S, Richardson A, Strong R (2011). Inducing autophagy by rapamycin before, but not after, the formation of plaques and tangles ameliorates cognitive deficits. PLoS ONE.

[34] Cortes CJ, Qin K, Cook J (2012). Rapamycin delays disease onset and prevents PrP plaque deposition in a mouse model of Gerstmann-Straussler-Scheinker disease. J Neurosci.

[35] Zhang X, Li L, Chen S (2011). Rapamycin treatment augments motor neuron degeneration in SOD1(G93A) mouse model of amyotrophic lateral sclerosis. Autophagy.

[36] Sarkar S, Ravikumar B, Floto RA (2009). Rapamycin and mTOR-independent autophagy inducers ameliorate toxicity of polyglutamine-expanded huntingtin and related proteinopathies. Cell Death Differ.

[37] Kim D, Nguyen MD, Dobbin MM (2007). SIRT1 deacetylase protects against neurodegeneration in models for Alzheimer's disease and amyotrophic lateral sclerosis. EMBO J.

[38] Khan RS, Fonseca-Kelly Z, Callinan C (2012). SIRT1 activating compounds reduce oxidative stress and prevent cell death in neuronal cells. Front Cell Neurosci.

[39] Frozza RL, Bernardi A, Hoppe JB (2013). Neuroprotective effects of resveratrol against abeta administration in rats are improved by lipid-core nanocapsules. Mol Neurobiol.

[40] Morselli E, Maiuri MC, Markaki M (2010). Caloric restriction and resveratrol promote longevity through the Sirtuin-1-dependent induction of autophagy. Cell Death Dis.

[41] Wang IF, Guo BS, Liu YC (2012). Autophagy activators rescue and alleviate pathogenesis of a mouse model with proteinopathies of the TAR DNA-binding protein 43. Proc Natl Acad Sci USA.

[42] Velloso NA, Dalmolin GD, Gomes GM (2009). Spermine improves recognition memory deficit in a rodent model of Huntington's disease. Neurobiol Learn Mem.

[43] Fernandez-Estevez MA, Casarejos MJ, Lopez Sendon J (2014). Trehalose reverses cell malfunction in fibroblasts from normal and Huntington's disease patients caused by proteosome inhibition. PLoS ONE.

[44] Zhang X, Chen S, Song L (2014). MTOR-independent, autophagic enhancer trehalose prolongs motor neuron survival and ameliorates the autophagic flux defect in a mouse model of amyotrophic lateral sclerosis. Autophagy.

[45] Rodriguez-Navarro JA, Rodriguez L, Casarejos MJ (2010). Trehalose ameliorates dopaminergic and tau pathology in parkin deleted/tau overexpressing mice through autophagy activation. Neurobiol Dis.

[46] Schaeffer V, Lavenir I, Ozcelik S (2012). Stimulation of autophagy reduces neurodegeneration in a mouse model of human tauopathy. Brain.

[47] Aguib Y, Heiseke A, Gilch S (2009). Autophagy induction by trehalose counteracts cellular prion infection. Autophagy.

[48] Karapetyan YE, Sferrazza GF, Zhou M (2013). Unique drug screening approach for prion diseases identifies tacrolimus and astemizole as antiprion agents. Proc Natl Acad Sci USA.

[49] Shahwan A, Farrell M, Delanty N (2005). Progressive myoclonic epilepsies: a review of genetic and therapeutic aspects. Lancet Neurol.

[50] Koskenkorva P, Khyuppenen J, Niskanen E (2009). Motor cortex and thalamic atrophy in Unverricht-Lundborg disease: voxel-based morphometric study. Neurology.

[51] Polajnar M, Zerovnik E (2011). Impaired autophagy: a link between neurodegenerative diseases and progressive myoclonus epilepsies. Trends Mol Med.

[52] Taylor TN, Greene JG, Miller GW (2010). Behavioral phenotyping of mouse models of Parkinson's disease. Behav Brain Res.

[53] Filali M, Lalonde R, Rivest S (2011). Anomalies in social behaviors and exploratory activities in an APPswe/PS1 mouse model of Alzheimer's disease. Physiol Behav.

[54] Menalled L, El-Khodor BF, Patry M (2009). Systematic behavioral evaluation of Huntington's disease transgenic and knock-in mouse models. Neurobiol Dis.

[55] Gupta S, Kulhara P (2010). What is schizophrenia: a neurodevelopmental or neurodegenerative disorder or a combination of both? A critical analysis. Indian J Psychiatry.

[56] Goodwin GM, Martinez-Aran A, Glahn DC (2008). Cognitive impairment in bipolar disorder: neurodevelopment or neurodegeneration? An ECNP expert meeting report. Eur Neuropsychopharmacol.

[57] Cannon TD, Thompson PM, van Erp TG (2002). Cortex mapping reveals regionally specific patterns of genetic and disease-specific gray-matter deficits in twins discordant for schizophrenia. Proc Natl Acad Sci USA.

[58] Merenlender-Wagner A, Malishkevich A, Shemer Z (2013). Autophagy has a key role in the pathophysiology of schizophrenia. Mol Psychiatry.

[59] Haack TB, Hogarth P, Kruer MC (2012). Exome sequencing reveals *de novo* WDR45 mutations causing a phenotypically distinct, X-linked dominant form of NBIA. Am J Hum Genet.

[60] Cleary C, Linde JA, Hiscock KM (2008). Antidepressive-like effects of rapamycin in animal models: implications for mTOR inhibition as a new target for treatment of affective disorders. Brain Res Bull.

[61] Kara NZ, Toker L, Agam G (2013). Trehalose induced antidepressant-like effects and autophagy enhancement in mice. Psychopharmacology.

[62] Sarkar S, Krishna G, Imarisio S (2008). A rational mechanism for combination treatment of Huntington's disease using lithium and rapamycin. Hum Mol Genet.

[63] Fornai F, Longone P, Cafaro L (2008). Lithium delays progression of amyotrophic lateral sclerosis. Proc Natl Acad Sci USA.

[64] Heiseke A, Aguib Y, Riemer C (2009). Lithium induces clearance of protease resistant prion protein in prion-infected cells by induction of autophagy. J Neurochem.

[65] Sarkar S, Floto RA, Berger Z (2005). Lithium induces autophagy by inhibiting inositol monophosphatase. J Cell Biol.

[66] Williams RS, Cheng L, Mudge AW (2002). A common mechanism of action for three mood-stabilizing drugs. Nature.

[67] Leng Y, Liang MH, Ren M (2008). Synergistic neuroprotective effects of lithium and valproic acid or other histone deacetylase inhibitors in neurons: roles of glycogen synthase kinase-3 inhibition. J Neurosci.

[68] Arico S, Petiot A, Bauvy C (2001). The tumor suppressor PTEN positively regulates macroautophagy by inhibiting the phosphatidylinositol 3-kinase/protein kinase B pathway. J Biol Chem.

[69] Gamez J, Salvado M, Martinez de la Ossa A (2013). Lithium for treatment of amyotrophic lateral sclerosis: much ado about nothing. Neurologia.

[70] Shimada K, Motoi Y, Ishiguro K (2012). Long-term oral lithium treatment attenuates motor disturbance in tauopathy model mice: implications of autophagy promotion. Neurobiol Dis.

[71] Li L, Zhang S, Zhang X (2013). Autophagy enhancer carbamazepine alleviates memory deficits and cerebral amyloid-beta pathology in a mouse model of Alzheimer's disease. Curr Alzheimer Res.

[72] Gao C, Cao W, Bao L (2010). Autophagy negatively regulates Wnt signalling by promoting Dishevelled degradation. Nat Cell Biol.

[73] Choi JD, Ryu M, Ae Park M (2012). FIP200 inhibits beta-catenin-mediated transcription by promoting APC-independent beta-catenin ubiquitination. Oncogene.

[74] Chang HW, Lee YS, Nam HY (2013). Knockdown of beta-catenin controls both apoptotic and autophagic cell death through LKB1/AMPK signaling in head and neck squamous cell carcinoma cell lines. Cell Signal.

[75] Inoki K, Ouyang H, Zhu T (2006). TSC2 integrates Wnt and energy signals *via* a coordinated phosphorylation by AMPK and GSK3 to regulate cell growth. Cell.

[76] Mao Y, Ge X, Frank CL (2009). Disrupted in schizophrenia 1 regulates neuronal progenitor proliferation *via* modulation of GSK3beta/beta-catenin signaling. Cell.

[77] Kim JY, Duan X, Liu CY (2009). DISC1 regulates new neuron development in the adult brain *via* modulation of AKT-mTOR signaling through KIAA1212. Neuron.

[78] Li X, Bijur GN, Jope RS (2002). Glycogen synthase kinase-3beta, mood stabilizers, and neuroprotection. Bipolar Disord.

[79] Hall AC, Brennan A, Goold RG (2002). Valproate regulates GSK-3-mediated axonal remodeling and synapsin I clustering in developing neurons. Mol Cell Neurosci.

[80] Toledo EM, Inestrosa NC (2010). Activation of Wnt signaling by lithium and rosiglitazone reduced spatial memory impairment and neurodegeneration in brains of an APPswe/PSEN1DeltaE9 mouse model of Alzheimer's disease. Mol Psychiatry.

[81] Purro SA, Dickins EM, Salinas PC (2012). The secreted Wnt antagonist Dickkopf-1 is required for amyloid beta-mediated synaptic loss. J Neurosci.

[82] Killick R, Ribe EM, Al-Shawi R (2014). Clusterin regulates beta-amyloid toxicity *via* Dickkopf-1-driven induction of the wnt-PCP-JNK pathway. Mol Psychiatry.

[83] Rawal N, Corti O, Sacchetti P (2009). Parkin protects dopaminergic neurons from excessive Wnt/beta-catenin signaling. Biochem Biophys Res Commun.

[84] Sharma J, Mulherkar S, Mukherjee D (2012). Malin regulates Wnt signaling pathway through degradation of dishevelled2. J Biol Chem.

[85] Carreira-Barbosa F, Concha ML, Takeuchi M (2003). Prickle 1 regulates cell movements during gastrulation and neuronal migration in zebrafish. Development.

[86] Chan DW, Chan CY, Yam JW (2006). Prickle-1 negatively regulates Wnt/beta-catenin pathway by promoting Dishevelled ubiquitination/degradation in liver cancer. Gastroenterology.

